# A determining factor for insect feeding preference in the silkworm, *Bombyx mori*

**DOI:** 10.1371/journal.pbio.3000162

**Published:** 2019-02-27

**Authors:** Zhong-Jie Zhang, Shuai-Shuai Zhang, Bao-Long Niu, Dong-Feng Ji, Xiao-Jing Liu, Mu-Wang Li, Hua Bai, Subba Reddy Palli, Chen-Zhu Wang, An-Jiang Tan

**Affiliations:** 1 Zhejiang Academy of Agricultural Sciences, Hangzhou, China; 2 Key Laboratory of Insect Developmental and Evolutionary Biology, Center for Excellence in Molecular Plant Sciences, Shanghai Institute of Plant Physiology and Ecology, Chinese Academy of Sciences, Shanghai, China; 3 State Key Laboratory of Integrated Management of Pest Insects and Rodents, Institute of Zoology, Chinese Academy of Sciences, Beijing, China; 4 Sericultural Research Institute, Jiangsu University of Science and Technology, Zhenjiang, China; 5 Department of Genetics, Development, and Cell Biology, Iowa State University, Ames, Iowa, United States of America; 6 Department of Entomology, University of Kentucky, Lexington, Kentucky, United States of America; Cornell University, UNITED STATES

## Abstract

Feeding preference is critical for insect adaptation and survival. However, little is known regarding the determination of insect feeding preference, and the genetic basis is poorly understood. As a model lepidopteran insect with economic importance, the domesticated silkworm, *Bombyx mori*, is a well-known monophagous insect that predominantly feeds on fresh mulberry leaves. This species-specific feeding preference provides an excellent model for investigation of host-plant selection of insects, although the molecular mechanism underlying this phenomenon remains unknown. Here, we describe the gene *GR66*, which encodes a putative bitter gustatory receptor (GR) that is responsible for the mulberry-specific feeding preference of *B*. *mori*. With the aid of a transposon-based, clustered regularly interspaced short palindromic repeats (CRISPR)/CRISPR-associated protein-9 nuclease (Cas9) system, the *GR66* locus was genetically mutated, and homozygous mutant silkworm strains with truncated gustatory receptor 66 (GR66) proteins were established. *GR66* mutant larvae acquired new feeding activity, exhibiting the ability to feed on a number of plant species in addition to mulberry leaves, including fresh fruits and grain seeds that are not normally consumed by wild-type (WT) silkworms. Furthermore, a feeding choice assay revealed that the mutant larvae lost their specificity for mulberry. Overall, our findings provide the first genetic and phenotypic evidences that a single bitter GR is a major factor affecting the insect feeding preference.

## Abstract 2

Genetic analysis of the silkworm Bombyx mori reveals that mutation of a single bitter gustatory receptor GR66 converts this typical monophagous insect, with its famous predilection for mulberry leaves, into a polyphagous species, providing novel insights into how insect feeding preference is determined.

## Author summary

The molecular mechanism underlying species-specific feeding preference in insects is poorly understood. The silkworm, *Bombyx mori*, is a typical monophagous plant-eating insect, but the genetic basis for its famous mulberry-specific feeding preference is unknown. Here, we identify gustatory receptor 66 (GR66) as a determinant of the silkworm’s mulberry-specific monophagy. GR66-mutant larvae generated by clustered regularly interspaced short palindromic repeats (CRISPR)/CRISPR-associated protein-9 nuclease (Cas9) acquired new feeding activity and showed the ability to feed on various plant species that are not normally consumed by the wild-type (WT) animals; a two-choice assay demonstrated that the mutant larvae had lost their feeding preference for mulberry. Our genetic and phenotypic evidence therefore demonstrates that GR66 is a major factor affecting the feeding preference of the silkworm.

## Introduction

Chemosensory processes, including olfaction and gustation, are critical for host-plant selection in phytophagous insects [1,2]. Olfaction is responsible for host orientation, and gustation plays a central role in host selection [3,4]. Insect gustatory receptors (GRs), as well as olfactory receptors (ORs), therefore play critical roles in determining insect feeding preference. Most insect GRs are expressed exclusively in gustatory receptor neurons (GRNs) and transmit signals through GRNs to regulate insect feeding behaviors [5,6]. Insect GRs are known to recognize sugars, bitter compounds, and nonvolatile pheromones [7,8]. In *Drosophila melanogaster*, GR5a and GR66a are found in different populations of GRNs [5]. GR5a-positive GRNs respond to various sugars, and GR66a-positive GRNs respond to many bitter compounds [9,10]. In the butterfly, *Papilio xuthus*, a GR was reported to be involved in host-plant recognition for oviposition [11]. In addition, GRs are also required for the detection of CO_2_, nutrients, light, and temperature [12–14]. Large numbers of insect GRs have been identified in many insect species [15–24]. However, most GRs have not been functionally characterized, and the roles played by these GRs in insect feeding preferences remain unclear.

Based on the host-plant selection range, the feeding preferences of phytophagous insects are classified as monophagous, oligophagous, and polyphagous. Lepidoptera, the largest lineage of phytophagous insects, includes many important agricultural and forest pests that exhibit high diversity in terms of feeding preference. The domesticated silkworm, *Bombyx mori*, is a beneficial lepidopteran insect that has been a major contributor to silk production for thousands of years. One of the main characteristics of *B*. *mori* is its monophagous feeding preference, and silkworm larvae predominantly feed on fresh mulberry leaves (*Morus alba* L.). Several polyphagous silkworm mutant strains that feed on the leaves of various plants that are rejected by normal silkworms have been reported [25,26]. Genetic analysis of one representative strain, Sawa-J, revealed that a major recessive gene on the *polyphagous* (*pph*) locus was potentially responsible for this change in feeding preference [27]. However, the molecular mechanism underlying the monophagous feeding preference of *B*. *mori* is unknown, and whether GR genes are involved the feeding preference of silkworm remains to be determined. Recently, a complete set of 76 GR genes was identified in *B*. *mori* [28]. Among these genes, only three sugar GRs were functionally characterized [29–31], whereas most of the GRs remained functionally identified, including 66 putative bitter GRs [28].

The biological functions of most insect GRs are poorly understood, especially those of nondrosophilid insects, due to the lack of reverse genetic approaches for the study of these insect species. This is especially true for lepidopteran species, because RNA interference functions with variable efficiency in many species [32]. Recent advances in the development of targeted genomic manipulation tools provide great benefits for functional genomic research of lepidopteran insects. These genomic manipulation tools—including zinc-finger nucleases (ZFNs), transcription activator-like effector nucleases (TALENs), and the clustered regularly interspaced short palindromic repeats (CRISPR)/CRISPR-associated protein-9 nuclease (Cas9) system—have been extensively used to generate targeted mutations at single or multiple sites in many organisms in vitro and in vivo [33–35]. Among these tools, the CRISPR/Cas9 system is the most extensively used mutagenesis system due to its high mutagenic efficiency and simple procedure. Among lepidopteran insects, the CRISPR/Cas9 system has been successfully established in *B*. *mori* [36–38], *Spodoptera litura* [39], *Plutella xylostella* [40], and *Helicoverpa armigera* [41].

In the current study, we investigated the genetic basis for the feeding preference towards mulberry exhibited by silkworm. We mutated the *GR66* gene, which encodes a putative bitter GR, in *B*. *mori* using the Cas9/small guide RNA (sgRNA) system. Homozygous *GR66* mutant larvae exhibited expanded diets, indicating that the *GR66* gene is responsible for mulberry-specific feeding behavior in the silkworm. Acquiring new feeding activity in the silkworm will contribute to modern sericulture as well as to the understanding of the molecular mechanisms of insect–host interactions.

## Results

### Tissue-specific expression and cell localization of *GR66*

**Fig 1:**
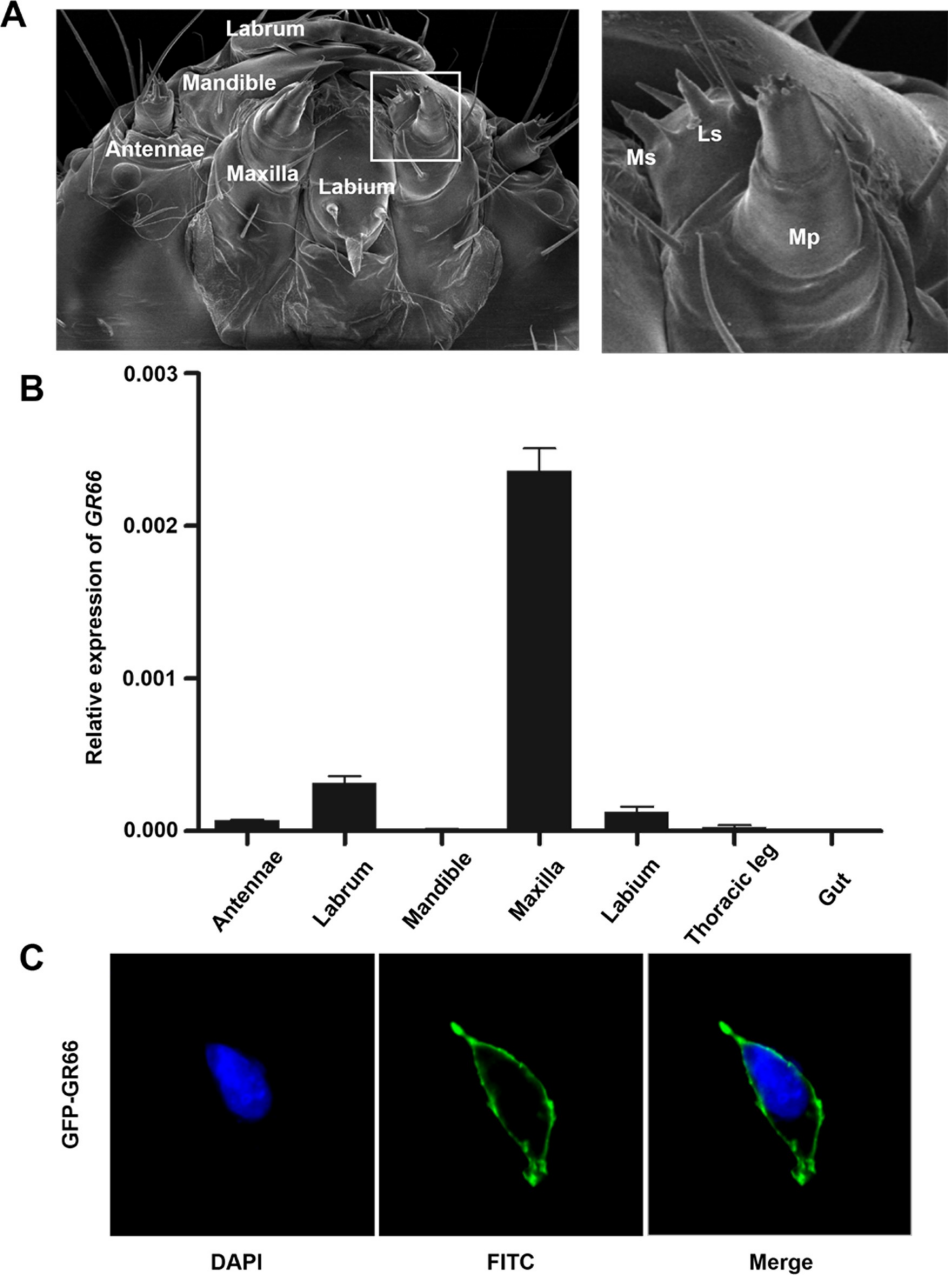
*GR66* expression in tissues and cellular localization of GR66. (A) Scanning electron micrographs of first-instar heads. The area inside the white box is enlarged on the right. (B) Relative mRNA levels of *BmGR66* in *Bombyx mori* tissues as determined by qRT-PCR. Total RNA was isolated from the antennae, labra, mandibles, maxillae, labia, thoracic legs, and midguts. The RNA was converted to cDNA, which was used as a template to quantify *BmGR66* mRNA levels using *Bmrp49* as a reference gene. The data shown was the mean ± SEM (*n* = 3). Underlying data can be found in S1 Data. (C) Photographs of HEK293T cells expressing the EGFP-GR66 fusion protein and stained with DAPI. *n* = 32. Scale bars: 10 μm. cDNA, complementary DNA; EGFP, enhanced green fluorescent protein; FITC, fluorescein isothiocyanate; GR66, gustatory receptor 66; HEK293T, human embryonic kidney 239T; Ls, lateral sensilla; Mp, maxillary palp; Ms, medial sensilla; qRT-PCR, quantitative real-time PCR; SEM, standard error of the mean.

**Fig 2:**
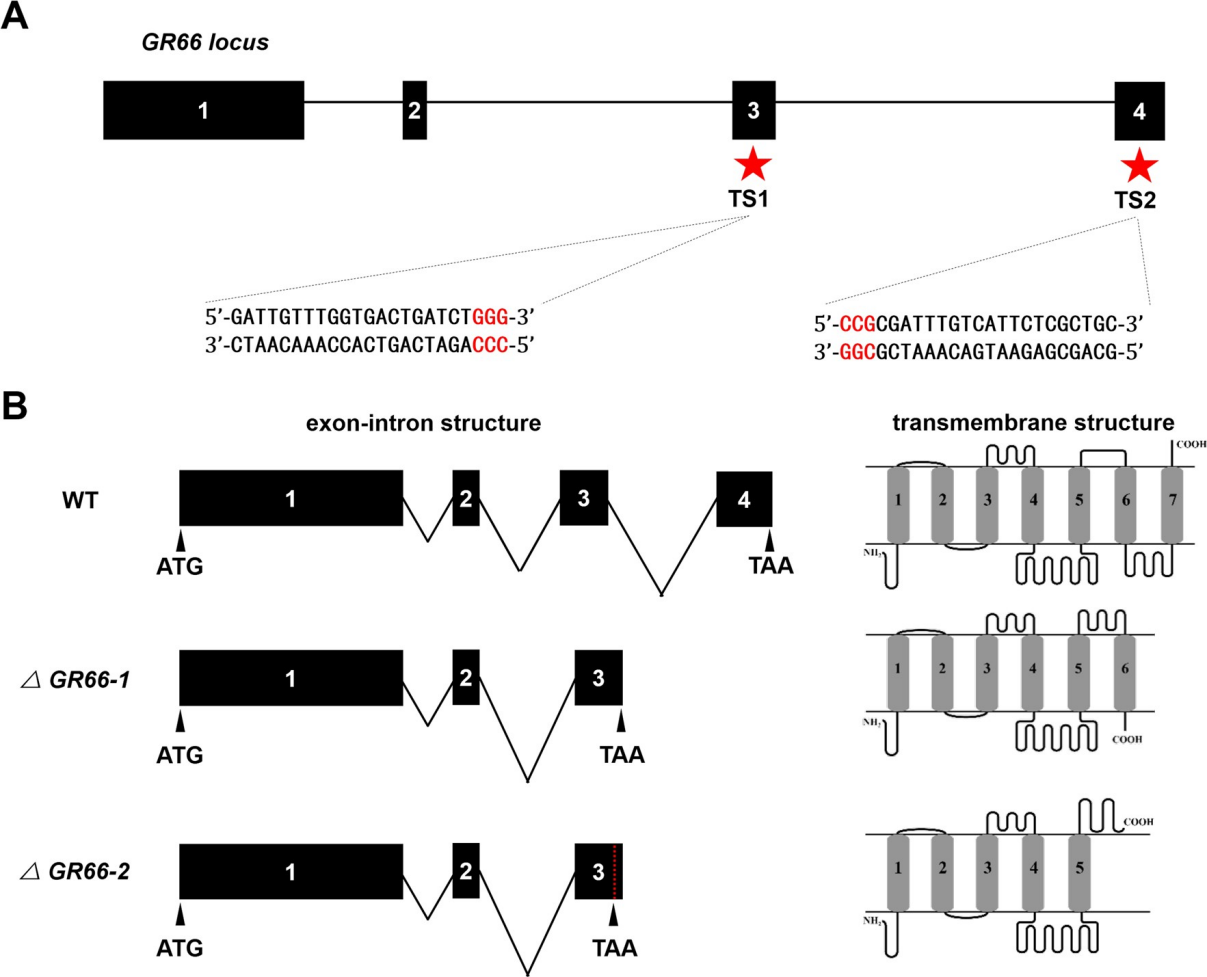
CRISPR/Cas9-mediated knockout and comparison among WT and mutants. (A) Schematic depiction of the *GR66* locus and sgRNA targeting sites. The sgRNA targeting sites, namely, TS1 and TS2, are located on the sense strand of exon-3 and the antisense strand of exon-4, respectively. The sgRNA targeting sequence is shown in black, and the PAM sequence is shown in red. (B) Comparison of gene structure among the WT and two homozygous mutant lines. Left, exon-intron structure of *GR66*. The 3ʹ fragment of exon 3, the third intron and the 5ʹ fragment of exon 4 were excised in both homozygous mutant lines. In *ΔGR66-2*, excision of the sequences caused frameshift mutations. The dotted red line indicates the premature termination codon. Right, transmembrane domain predictions of GR66 for the WT and two homozygous mutant lines. In WT, the GR66 protein consists of seven transmembrane domains, an intracellular N terminus and an extracellular C terminus. In *ΔGR66-1*, the truncated protein consists of six transmembrane domains, and the N terminus and C terminus are both extracellular. In *ΔGR66-2*, the truncated protein consists of only five transmembrane domains. The orientation of the N terminus and C terminus are the same in WT. CRISPR/Cas9, clustered regularly interspaced short palindromic repeats/CRISPR-associated protein-9 nuclease; GR66, gustatory receptor 66; PAM, protospacer adjacent motif; sgRNA, small guide RNA; WT, wild type.

It was reported that there are 76 putative GRs distributed on 16 of the 28 chromosomes of *B*. *mori* [28]. Among these genes, only one putative bitter GR gene, *GR66*, was identified as being located on the third chromosome. The genomic locus of this gene is within the putative *pph* locus of the polyphagous Sawa-J silkworm strain [27]. This finding indicates that *GR66* might be the candidate gene for the *pph* locus and could be involved in the feeding preference of silkworm. We first investigated the relative mRNA levels of *GR66* in different larval tissues using quantitative real-time PCR (qRT-PCR). It has been reported that most insect GRs are localized in the taste sensilla of the larval mouthparts [28,42] (Fig 1A). As expected, *GR66* was predominantly expressed in larval maxillae (Fig 1B). The open reading frame (ORF) of the *GR66* gene contains 1,140 base pairs and encodes a 380-amino-acid polypeptide. Bioinformatic analysis revealed that the GR66 protein consists of seven transmembrane domains with an intracellular N terminus, which is distinct from the structures of members of the G-protein-coupled receptor (GPCR) family (Fig 2B). We further investigated the cellular localization of this protein via transfection of an enhanced green fluorescent protein (EGFP)-fused GR66 expression plasmid into mammalian 293T cells. The results showed that the protein is localized on the cell membrane (Fig 1C).

### Establishment of *GR66* homozygous mutant lines

To investigate the potential involvement of *GR66* in the feeding preference of silkworm, we genetically ablated *GR66* using a transposon-based, Cas9/sgRNA-mediated mutagenesis system [37]. Two independent transgenic lines were established by transposon-mediated germline transformation. One transgenic line expressed Cas9 under the control of the germ-cell–specific promoter *Bmnos* [37], and the other line expressed two sequence-specific sgRNAs targeting *GR66* (Fig 2A) under the control of the *BmU6* promoter [38]. Each line also expressed an IE1 promoter-derived fluorescent marker (EGFP in the Cas9-expressing line or DsRed2 in the sgRNA-expressing line) to facilitate the screening of positive individuals from the embryonic stage [37]. In the F1 hybrids between the Cas9 and sgRNA lines, somatic mutagenesis was identified by PCR-based analysis and subsequent sequencing. Mutants were generated at a single site or both sites (S1A Fig), indicating that successful mutagenesis was induced by the transgenic CRISPR/Cas9 system. Somatic mutants of *GR66* showed no deleterious phenotype compared with the wild-type (WT) animals, indicating that knocking out *GR66* did not interfere with silkworm development and fertility. To obtain heritable, nontransgenic, homologous mutants to assess feeding preference, a series of crossing strategies and PCR-based screening experiments were performed (S1B Fig) as described previously [43]. Finally, two independent homozygous lines with truncated GR66 proteins were established (S2 Fig). One mutant line (*ΔGR66-1*) had a 929-bp genomic DNA deletion at the *GR66* locus, resulting in a 180-bp deletion in the ORF and to a truncated 319-aa protein, which was 60 aa shorter than WT GR66 protein (S2 Fig). The other mutant line (*ΔGR66-2*) had a 931-bp genomic deletion at the *GR66* locus, resulting in a 182-bp deletion in the ORF and to a 312-aa protein, which was 67 aa shorter than the WT GR66 protein (S2 Fig). The truncated GR66 proteins of the *ΔGR66-1* and *ΔGR66-2* mutants contained only six or five transmembrane domains, respectively (Fig 2B). Because the truncated proteins did not have all seven transmembrane domains that are essential for the function of the membrane proteins [44,45], we presumed that both mutants lacked *GR66* functions.

### Feeding behavior in *GR66* mutant silkworms

**Fig 3:**
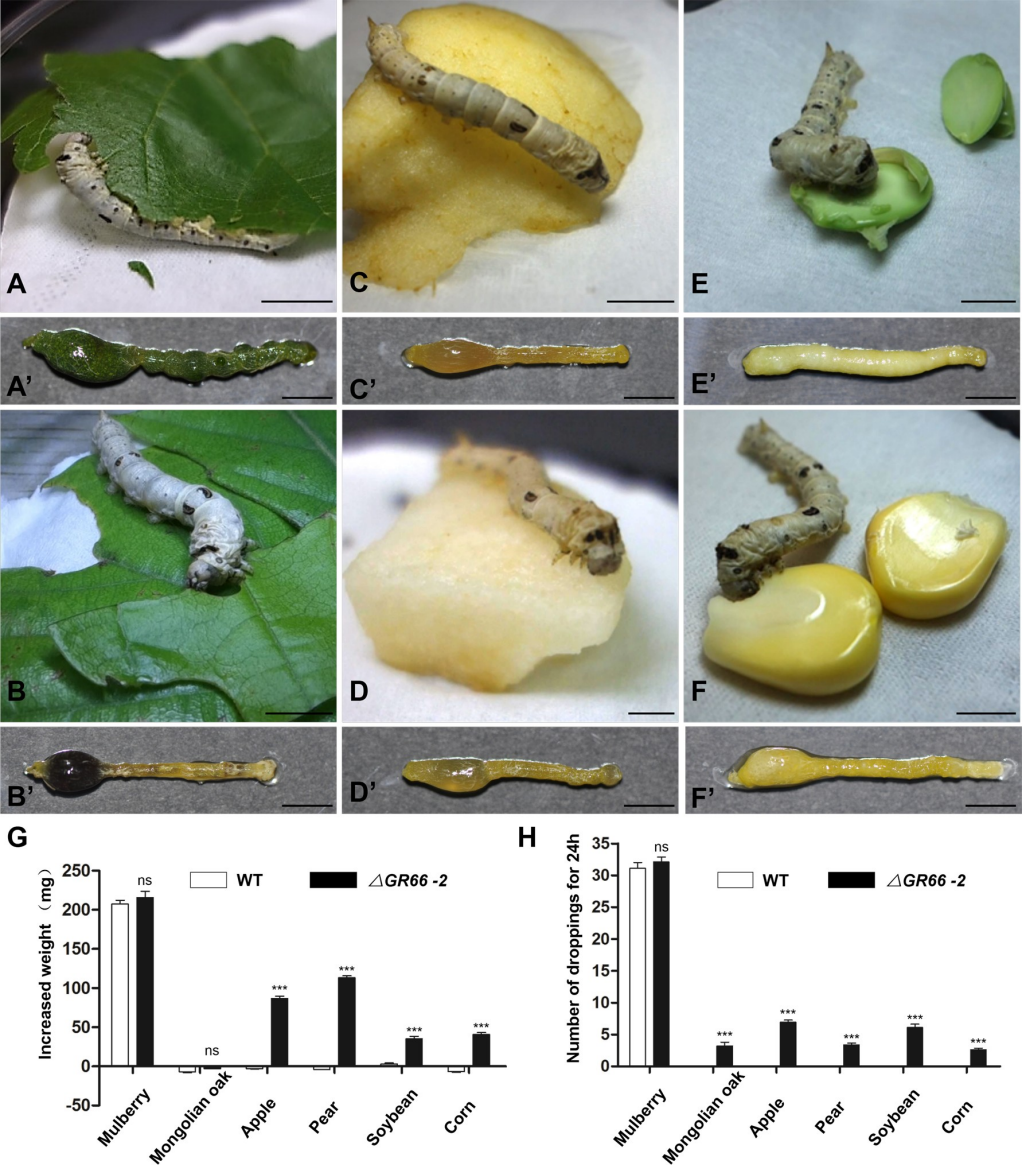
The feeding preference of the *ΔGR66-2* line was expanded. The newly moulted fifth-instar larvae of *ΔGR66-2* after 24 h of starvation ate mulberry leaves (A), Mongolian oak (B), apple (C), pear (D), soybean (E), and corn (F). (G) *ΔGR66-2* fed on apple, pear, soybean, and corn showed a significant increase in weight when compared to WT animals fed on the same materials. The midguts of *ΔGR66-2* after 24 h of starvation showed that the animals ate mulberry leaves (A’), Mongolian oak (B’), apple (C’), pear (D’), soybean (E’), and corn (F’). Scale bars: 50 mm in A, A’, B, B’, C, C’, D, D’, E, E’, F, and F’. (G) *ΔGR66-2* fed on apple, pear, soybean, and corn showed a significant increase in weight when compared to WT animals fed on the same materials. The WT and *ΔGR66-2* larvae fed on Mongolian oak did not show an increased body weights after 24 h of feeding. (H) Number of droppings (per larva) from larvae fed on mulberry, Mongolian oak, apple, pear, soybean, and corn at 24 h after initiation of feeding. The data shown were the mean ± SEM (*n* = 18 silkworms). The asterisks indicated significant differences as calculated by a two-tailed *t*-test: ns (not significant), ***P < 0.001. Underlying data can be found in S1 Data. GR66, gustatory receptor 66; SEM, standard error of the mean.

Consistent with the transgenic somatic mutants, homologous *GR66* mutant silkworms were fully viable and fertile. We first used homozygous *ΔGR66-2* newly moulted fifth-instar larvae to assess feeding behavior. After 24 h of starvation treatment to facilitate feeding sensitivity, both WT and homozygous *GR66-2* mutant larvae were provided various food sources for 24 h (Fig 3A–3E), and then, the increase in weight and number of droppings were recorded (Fig 3G and 3H). The leaves of Mongolian oak (*Quercus mongolica* Fisch. ex Ledeb.), fruits of apple (*Malus domestica*) and pear (*Pyrus* spp.), and seeds of soybean (*Glycine max*) and corn (*Zea mays*) were subjected to analysis. Mulberry leaves were also used as a control. Both WT and mutant larvae ate the mulberry leaves and exhibited normal development (Fig 3A and S1 Movie). Leaves of Mongolian oak are known food sources of Chinese oak silkworm, *Antherea pernyi*, but are not consumed by *B*. *mori*. The *ΔGR66-2* larvae ate the oak leaves (Fig 3B and S2 Movie), and droppings were observed (Fig 3H), but the body weights did not increase significantly (Fig 3G). The *ΔGR66-2* larvae exhibited a 15.96% weight increase with approximately seven droppings per larva after feeding on apple, whereas the WT animals did not attempt to consume apple, and no droppings were observed (Fig 3C, 3G and 3H and S3 Movie). Furthermore, we found that the *ΔGR66-2* larvae could also feed on pear (Fig 3D and S4 Movie), which belongs to the same family as apple, namely, Rosaceae. A 25.47% weight increase was observed for *ΔGR66-2* larvae, whereas no significant increase was observed for WT animals (Fig 3G and 3H). The *ΔGR66-2* larvae could feed on both fresh soybean and corn, with a 10.56% and 14.08% increase in weight, respectively, whereas no significant weight increase was observed for WT animals (Fig 3E–3H, S5 Movie and S6 Movie). After feeding, the larvae were dissected to confirm food digestion, and the results showed that the midguts were filled with the residues of the indicated foods (Fig 3A’–3F’). Additionally, the Mongolian oak leaf residue diffused into the anterior part of the midguts (Fig 3B’), indicating that Mongolian oak leaves could not be digested well. This finding also explained why the body weight did not increase significantly (Fig 3G). A similar result was obtained when the *ΔGR66-*1 mutant line was subjected to analysis (S3 Fig). Notably, none of the larvae could survive the entire fifth-instar stage when reared on food other than mulberry (S4 Fig), indicating that *B*. *mori* mostly adapted to mulberry leaves during long-term cultivation.

### Feeding preference of *GR66* mutants

**Fig 4:**
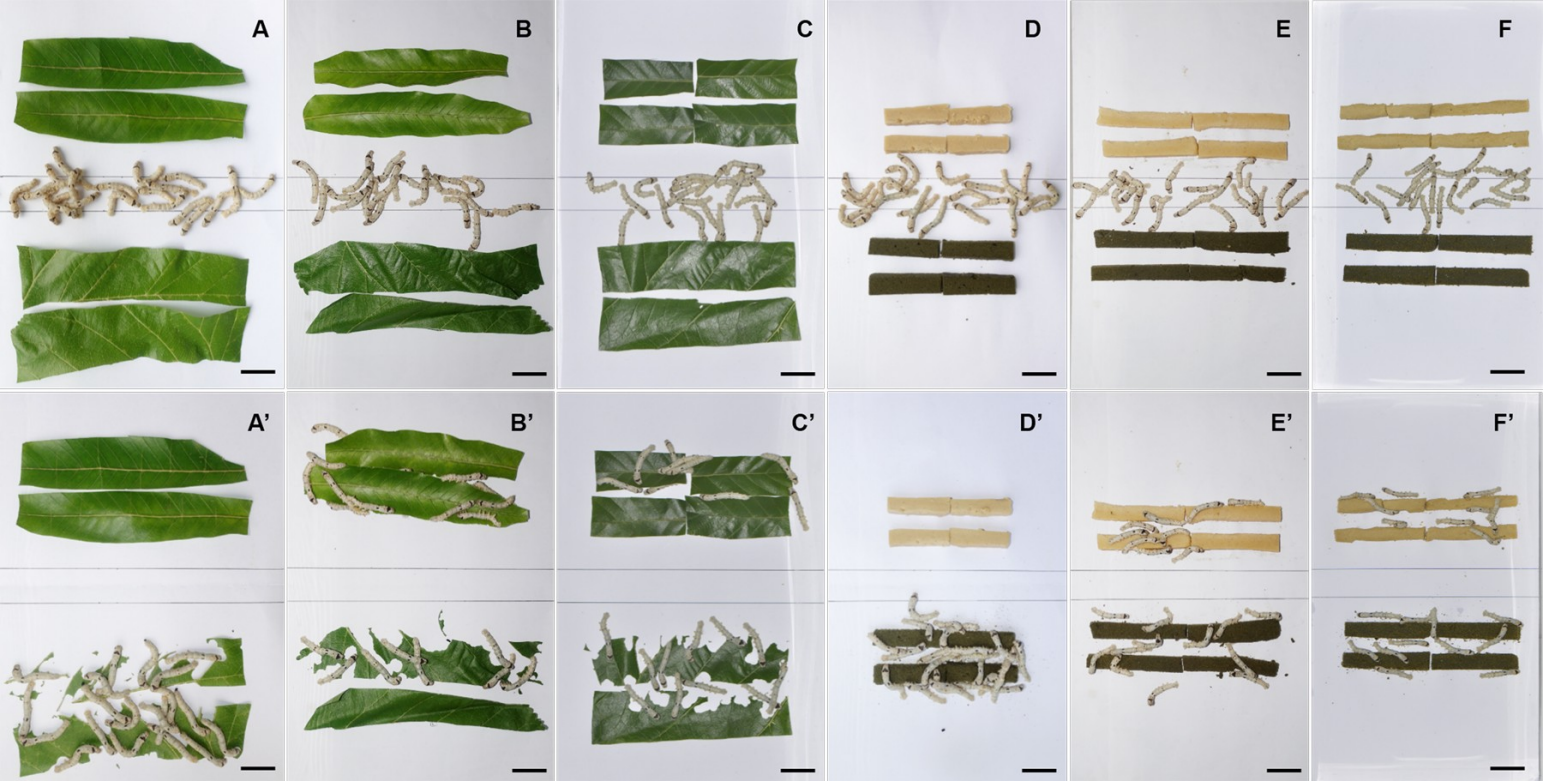
Two-choice assays with newly moulted fifth-instar larvae. The newly moulted fifth-instar larvae of WT after 24 h of starvation released between mulberry leaves and Mongolian oak leaves after 0 (A) and 1 h (A’). The newly moulted fifth-instar larvae of *ΔGR66-2* after 24 h of starvation released between mulberry leaves and Mongolian oak leaves after 0 (B) and 1 h (B’). The newly moulted fifth-instar larvae of *ΔGR66-1* after 24 h of starvation released between mulberry leaves and Mongolian oak leaves after 0 (C) and 1 h (C’). The newly moulted fifth instar larvae of WT after 24 h of starvation released between an artificial diet with mulberry leaf powder and an artificial diet with a 1:1 ratio of soybean powder to corn powder after 0 (D) and 1 h (D’). The newly moulted fifth instar larvae of *ΔGR66-2* after 24 h of starvation released between an artificial diet with mulberry leaf powder and an artificial diet with a 1:1 ratio of soybean powder to corn powder after 0 (E) and 1 h (E’). The newly moulted fifth-instar larvae of *ΔGR66-1* after 24 h of starvation released between an artificial diet with mulberry leaf powder and an artificial diet with a 1:1 ratio of soybean powder to corn powder after 0 (F) and 1 h (F’). Scale bars: 20 mm in A, A’, B, B’, C, C’, D, D’, E, and E’. Each assay was performed in triplicate (technical replicates). WT, wild-type.

To further investigate the feeding preference of *GR66* mutants, we performed a two-choice assay in prestarved fifth-instar larvae. Given a choice between mulberry leaves and Mongolian oak leaves, the WT larvae exhibited a strong preference for mulberry leaves and did not attempt to eat Mongolian oak leaves (Fig 4A and 4A’). In contrast, the *ΔGR66* larvae exhibited similar feeding preferences for both mulberry leaves and Mongolian oak leaves (Fig 4B, 4B’, 4C and 4C’). In addition, a commercial artificial diet containing mulberry leaf powder and another artificial diet that lacked mulberry leaf (1:1 ratio of soybean powder to corn powder) were also used for a two-choice assay. Similar to the previous result, the WT larvae exhibited a strong preference for the artificial diet containing mulberry (Fig 4D and 4D’), whereas the *ΔGR66* larvae exhibited similar feeding preferences for both artificial diets (Fig 4E, 4E’, 4F and 4F’). These results revealed that the *GR66* mutant larvae had lost their specificity for mulberry, suggesting that *GR66* is required for the mulberry-specific feeding preference of *B*. *mori*. In addition, we performed two-choice feeding assays with neonate larvae. Both the WT and *GR66* mutant neonate larvae exhibited a strong preference for the artificial diet containing mulberry (S5 Fig). Although this phenotypic consequence remained to be elucidated, we speculated that food choice of neonate larvae are also strongly affected by ORs, because olfaction is responsible for host orientation [46].

### Response of *GR66* mutants to sweet and bitter stimuli

Most insect GRs are located in the taste sensilla of the larval mouthparts, and it has been reported that the medial sensilla are responsible for sweet taste perception and lateral sensilla are responsible for bitter taste perception in Lepidoptera [28]. To investigate whether *GR66* mutants exhibit altered responses to different tastes, electrophysiological recording analysis on contact chemosensilla was performed on taste sensilla, including the medial and lateral styloconic sensilla of fifth-instar larvae in the *ΔGR66-2* line. We first investigated two sweet stimulants, namely, sucrose and myo-inositol, in the lateral sensilla. No difference was detected between WT and *GR66* mutants at a concentration of 10 mM, indicating that *GR66* depletion was irrelevant for the perception of these two sweet stimuli (S6A and S6B Fig). We subsequently investigated two bitter substances, namely, caffeine and salicin, in the medial sensilla at a concentration of 10 mM. The results showed that the electrophysiological response to these two substances was not affected by *GR66* depletion (S6C and S6D Fig). We further tested the response to caffeine and salicin at different concentrations, and similar results were obtained (S6E and S6F Fig). These results indicated that the *GR66* mutants did not exhibit altered responses to these typical sweet or bitter substances. Other compounds in mulberry leaves, especially the potential ligands of GR66, remain to be identified.

## Discussion

Molecular mechanisms of host-plant selection in phytophagous insects remain to be elucidated, and how GRs are involved in their feeding behaviors is poorly understood. To reveal the molecular mechanism underlying mulberry-specific herbivory in *B*. *mori*, we genetically ablated a putative bitter GR, *GR66*, via Cas9/sgRNA-mediated targeted mutagenesis. Homologous mutant larvae exhibited loss of mulberry specificity and the ability to feed on a wide range of food sources, indicating that *GR66* is a determinant of the monophagous feeding preference of *B*. *mori*.

Increasing numbers of insect GRs have been identified, and their critical roles in detection of environmental stimulations have been reported [7–14]. In phytophagous insects, most reported GRs belong to putative bitter GR subfamily and they are necessary in the recognition of many plant secondary metabolites, which are normally bitter compounds [47]. In *B*. *mori*, the subfamily of the bitter GRs contains up to 66 genes and is the largest subfamily among the total 76 identified GRs in *B*. *mori* [28]. None of these putative bitter GRs had been functionally elucidated until the current study on GR66. Our data strongly suggest that *GR66* is a major factor affecting the feeding preference of silkworm, because mutation of this gene could change the mulberry-specific herbivory of silkworm. We speculate that GR66 may serve as a feeding inhibitor in *B*. *mori*. This finding explains why GR66 mutagenesis could result in the acceptance of an expanded range of host-plant materials by the larvae. In WT animals, GR66 is active and inhibits the feeding behavior on nonhost materials, whereas certain compounds in mulberry leaves directly or indirectly repress GR66 activity, leading to initiation of such feeding behavior. Future validation of potential ligands of GR66 in mulberry leaves and identification of food components that dictate host specificity will be critical for elucidation of this species-specific feeding preference. In the current study, the *ΔGR66* strains did not exhibit significant electrophysiological differences in the selection of sweet or bitter substances, including salicin. Our results were different from previously reported results for the polyphagous silkworm strain Sawa-J, which exhibited reduced sensitivity to the bitter compound salicin [26]. Because the *pph* locus in the Sawa-J strain has not been mapped to a single gene [26], the different electrophysiological phenotypes between the Sawa-J and *ΔGR66-2* strains indicated that the putative involvement of different or additional genes, such as the many other GR genes in *B*. *mori*, should be taken into account to explain the monophagous feeding preference for mulberry. We presumed that the effects of these genes led to the Sawa-J strains and *GR66* mutants exhibiting different responses to salicin. Additionally, it is possible that *GR66* mutagenesis did not create completely null mutants (Fig 2), and truncated GR66 may still respond to salicin.

Mulberry leaves have been used as the only food source for mass rearing of silkworm for thousands of years. Due to limitations associated with labor and land consumption and seasonal cycles in the harvesting of fresh mulberry leaves, the development of silkworm strains that can feed on cost-effective diets instead of mulberry leaves has been pursued. Conversion of the monophagous silkworm to a polyphagous species by GR mutagenesis therefore provides a promising approach for the development of alternative food sources for mass rearing of silkworm. Furthermore, lepidopteran insects include a large number of agricultural and forest pests that exhibit high diversity in terms of feeding habits. Orthologous genes of *GR66* or other GRs in lepidopteran insects may play key roles in the species-specific feeding preferences of these insects. Insect feeding preference is a very complicated biological process and is probably more complex than determined by a single gene. Large numbers of insect GRs remains to be functional elucidated, and they should also be considered to play important roles in feeding preference. Elucidation of the critical role of GRs in insect feeding preference will provide insights into the mechanisms underlying insect feeding behavior and insect–plant interactions, facilitating the development of novel strategies for pest management.

## Materials and methods

### Silkworms

A multivoltine and monophagous silkworm strain, Nistari, was used in all the experiments. Larvae were fed fresh mulberry leaves at 25°C under standard conditions [48].

### Scanning electron microscopy

Heads were excised from newly hatched first-instar larvae of *B*. *mori*. The excised heads were washed in PBS and fixed with FAA solution (1:1:18 ratio of 37% to 40% formaldehyde to acetic acid anhydride to 50% ethanol). The fixed samples were dehydrated via exposure to gradually increasing concentrations of ethyl alcohol (50%, 60%, 70%, 80%, 90%, 95%, 100%) using a rotary machine. The heads were dried in a critical-point dryer and then coated with platinum prior to observation under a scanning electron microscope (JEOL).

### qRT-PCR

Total RNA was isolated from the antennae, labra, mandibles, maxillae, labia, thoracic legs, and midguts of third-day fifth-instar (L5D3) larvae using TRIzol reagent (Invitrogen). The RNA was treated with DNase I (Invitrogen) to remove genomic DNA. One microgram of total RNA was used to synthesize cDNA using the ReverAid First Strand cDNA Synthesis Kit (Fermentas). Relative mRNA levels were determined by qRT-PCR using SYBR Green real-time PCR master mix (TOYOBO). The PCR conditions used were as follows: initial incubation at 95°C for 1 min, followed by 40 cycles of 95°C for 15 s and 60°C for 1 min. The primers used for qRT-PCR are listed in S1 Table. Another primer pair—namely, RP49-F and RP49-R (S1 Table)—was used as an internal control [48].

### Molecular cloning and plasmid construction

The ORF of *BmGR66* was PCR-amplified using cDNA synthesized from the total RNA isolated from the maxillae at L5D3 as a template. The PCR products obtained were directly cloned into the pcDNA-3.0 vector to generate GR66-pcDNA3.0. To detect the expression of *BmGR66* in human embryonic kidney 293T (HEK293T) cells, the ORF of GFP was cloned and incorporated in-frame upstream of *BmGR66* with a flexible linker modifying the amino acids GGGGS. To construct the transgenic CRISPR/Cas9 system, we used the activator line *pBac[IE1-DsRed2-Nos-Cas9]* (*Nos-Cas9*), in which Cas9 was driven by a germ-cell–specific promoter, as described previously [37]. The plasmid *pBac[IE1-EGFP-U6-BmGR66-sgRNA]* (*U6-sgRNA*), used to express the sgRNA, was constructed as described previously [38]. The sgRNA targeting sites were designed as GN19NGG. The primers used for plasmid construction are listed in S1 Table.

### Cell culture and transfection

HEK293T cells were cultured in Dulbecco's modified Eagle’s medium (DMEM, Thermo Fisher Scientific) supplemented with 10% fetal bovine serum (FBS) at 37°C and 5% CO_2_. For receptor localization analysis, HEK293T cells were seeded in 35-mm sterilized glass-bottom dishes and incubated for 24 h. EGFP-GR66-pcDNA3.0 was transfected into HEK293T cells using Lipofectamine 2000 (Invitrogen). After 24 h, the cells were fixed with 4% paraformaldehyde for 15 min and finally incubated with DAPI for 10 minutes. The cells were visualized by fluorescence microscopy on a Zeiss LSM 510 confocal laser scanning microscope attached to a Zeiss Axiovert 200 microscope using a Zeiss Plan-Apochromat 63×/1.40 NA oil immersion lens.

### Germline transformation and mutagenesis analysis

Germline transformation of silkworm was performed as described previously [48]. For the transgenic CRISPR/Cas9 system, the *Nos-Cas9* line was crossed with the *U6-sgRNA* line, and genomic DNA was extracted from the *Nos-Cas9*:U6-sgRNA as previously described [38]. Subsequently, genomic PCR followed by sequencing was carried out to identify *GR66* mutant alleles.

### Screening strategy and establishment of homozygous mutant strains

To establish a stable homozygous mutant line, the *Nos-Cas9*:*U6-sgRNA* (F1) were crossed with the WT. For the F2 progeny that lacked fluorescence, PCR-based genotyping was performed using genomic DNA extracted from adult legs as templates. Removal of legs did not interfere with moth survival and fertility. Details regarding the crossing procedure are shown in S1 Fig. Briefly, we backcrossed F1 somatic mutants with WT moths and used PCR to identify heterozygous F2 mutant animals. The selected F2 mutants were backcrossed with WT moths again. The progeny of this cross were approximately 50% heterozygotes and 50% WT animals. The F3 heterozygous animals were then sib-mated. The progeny of this cross were approximately 25% homozygous mutants, 50% heterozygous mutants, and 25% WT animals. The F4 homozygous mutants were then sib-mated to obtain 100% homozygous animals, which were used in subsequent experiments.

### Larval feeding behavior assay

Newly moulted fifth-instar larvae were starved for 24 h prior to conducting the behavioural assay. After starvation, each larva was placed in a sterile culture dish separately. Different plant-derived food materials, such as mulberry (*M*. *alba*), Mongolian oak (*Q*. *mongolica* Fisch. ex Ledeb.), apple (*M*. *domestica)*, pear (*Pyrus* spp.), soybean (*G*. *max*), and corn (*Z*. *mays*), were placed in the culture dishes. After 24 h, the weights of larvae were recorded, and the number of droppings was counted. Two-choice feeding preference tests were performed using plant leaves or artificial diets. Leaves of mulberry and Mongolian oak were placed on separate sides of the container 2 cm away from the middle. A two-choice feeding assay with an artificial diet containing mulberry leaf powder and an artificial diet that was 1:1 ratio of soybean powder to corn powder was performed as described above. Twenty newly moulted fifth-instar larvae after starvation for 24 h or a brood of neonate larvae were placed in the center. Photographs were taken at 0 and 60 min after release.

### Electrophysiological recording

Tip recordings for insect contact chemosensilla were performed on the medial and lateral styloconic sensilla of fifth-instar *B*. *mori* larvae as described previously with some modification [49, 50]. Heads with the first thoracic segments were cut from newly hatched fifth-instar larvae that were starved for 24 h. An AgCl-coated silver loop was inserted into each head until pressure caused the mouthparts to open, and then the loop was connected to a copper miniconnector, which served as the recording electrode. A recording glass electrode filled with the stimulus solution was brought in contact with the tip of the styloconic sensillum under a dissecting microscope. Responses were recorded from both the medial and lateral styloconic sensilla on both sides of the head. Stimuli lasted 1 s and were separated by an interval of 3 min to allow for recovery and to minimize adaptation. The tip diameter size of the stimulating electrode was approximately 50 μm, which is suitable for stimulation of single styloconic sensilla. Action potentials (spikes) generated during the first second after stimulus onset were amplified by the amplifier (Syntech Taste Probe DTP-1; Hilversum, the Netherlands) and filtered (A/D-interface, Syntech IDAC-4; Hilversum, the Netherlands). The electrophysiological signals were recorded and analyzed with the aid of spike analysis programs for insect data (SAPID) Tools software, version 16.0 [51], as well as Autospike version 3.7 software (Syntech, Hilversum, the Netherlands). Solutions of sucrose, myo-inositol, caffeine, and salicin dissolved in 2 mM KCl were used as stimulants in the electrophysiological experiments. For each stimulant and corresponding sensillum responsive to the stimulant, 15 WT and mutant larvae that hatched from 3 to 5 different rearing batches were tested. A solution of 2 mM KCl served as a control. Data are presented as the means ± standard error of the means (SEMs).

### Statistical analysis

All the experiments in this study were performed with at least three replicates. All the data are expressed as the mean ± SEM. The differences between groups were examined by either two-tailed Student *t*-test or two-way ANOVA. Statistically significant differences are indicated by asterisks.

## Supporting information

S1 Fig Deletions in GR66 caused by CRISPR/Cas9 and an experimental diagram for generation of the homozygous mutant lines. (A) Genomic mutagenesis induced by the transgenic CRISPR/Cas9 system. Various deletion mutations of TS1 and TS2 were detected in heterozygous Nos-Cas9:U6-sgRNA offspring. The numbers in brackets in the middle of each sequence refer to the 1,403-bp interspace fragment that was found between the targeting sites. The PAM sequence is shown in red. (B) The strategy for generation of a homozygous mutant using the transgenic CRISPR/Cas9 system. (1) Preblastoderm silkworm embryos were injected with the transgenic plasmids *Nos-Cas9* or *U6-sgRNA* to produce two transgenic silkworm lines. (2) Subsequently, the two transgenic lines were hybridized to produce founder animals (F1), which expressed both Cas9 and *GR66* sgRNAs. (3) The F1 somatic mutant was backcrossed with WT to obtain F2 progeny. The F2 progeny that lacked fluorescence and complete deletion events were backcrossed with WT moths again to obtain F3 animals that were 50% heterozygotes and 50% WT animals. (4) The F3 heterozygous animals were then sib-mated to obtain F4 hybrids that were 25% F4 homozygous mutants, 50% heterozygous mutants, and 25% WT animals. (5) The F4 homozygous mutants were then sib-mated to obtain 100% homozygous F5 progeny, which were used in subsequent experiments. Two GR66 allele mutant lines were established. The sequence below shows the mutation event. The PAM sequence is shown in red. CRISPR/Cas9, clustered regularly interspaced short palindromic repeats/CRISPR-associated protein-9 nuclease; GR66, gustatory receptor 66; *Nos-Cas9*, *pBac[IE1-DsRed2-Nos-Cas9]*; PAM, protospacer adjacent motif; sgRNA, small guide RNA; *U6-sgRNA*, *pBac[IE1-EGFP-U6-BmGR66-sgRNA]*; WT, wild-type.
(TIF)

S2 Fig Comparison of *GR66* among WT, *ΔGR66-1*, and *ΔGR66-2*. (A) Genomic PCR of *GR66* of WT, *ΔGR66-1*, and *ΔGR66-2*. *ΔGR66-1* had a 929-bp genomic DNA deletion, and *ΔGR66-2* had a 931-bp genomic DNA deletion at the *GR66* locus. (B) RT-PCR of *GR66* of WT, *ΔGR66-1*, and *ΔGR66-2*. *ΔGR66-1* had a 180-bp deletion, and *ΔGR66-2* had a 182-bp deletion in the ORF. (C) Amino acid sequence alignment of the GR66 protein in WT, *ΔGR66-2*, and *ΔGR66-2*. ΔGR66-1 is 60 aa shorter than the WT GR66 protein. ΔGR66-2 is 67 aa shorter than the WT GR66 protein. Identical amino acids are indicated with “*.” GR66, gustatory receptor 66; ORF, open reading frame; RT-PCR, reverse transcription-PCR; WT, wild-type.
(TIF)

S3 Fig The feeding preference of the *ΔGR66-1* line was expanded. The newly moulted fifth-instar larvae of *ΔGR66-1* after 24 h of starvation ate mulberry leaves (A), Mongolian oak (B), apple (C), pear (D), soybean (E), and corn (F). (G) *ΔGR66-1* fed on apple, pear, soybean, and corn showed a significant increase in weight when compared to WT fed on the same materials. Scale bars: 5 mm in A, B, C, D, E, and F. (G) *ΔGR66-1* fed on apple, pear, soybean and corn showed a significant increase in weight when compared to WT fed on the same materials. The body weights of the larvae of WT and *ΔGR66-1* fed on Mongolian oak did not show an increase in weight after 24 h of feeding. (H) Number of droppings (per larva) from larvae fed on mulberry, Mongolian oak, apple, pear, soybean, and corn at 24 h after initiation of feeding. The data shown was the mean ± SEM (*n* = 18 silkworms). The asterisks indicated significant differences as calculated by a two-tailed *t*-test: ns (not significant), ***P < 0.001. Underlying data can be found in S1 Data. GR66, gustatory receptor 66; SEM, standard error of the mean; WT, wild-type.
(TIF)

S4 Fig Survival assays with *Δ*GR66-2 mutant neonate larvae. The data shown was the mean ± SEM (*n* = 30 silkworms). Each assay was performed in triplicate. Underlying data can be found in S1 Data. GR66, gustatory receptor 66; SEM, standard error of the mean.
(TIF)

S5 Fig Two-choice assays with neonate larvae. The neonate larvae of WT released between the artificial diet with mulberry leaf powder and the artificial diet with a 1:1 ratio of soybean powder to corn powder after 0 (A) and 1 h (A’). The neonate larvae of *ΔGR66-1* released between the artificial diet with mulberry leaf powder and the artificial diet with a 1:1 ratio of soybean powder to corn powder after 0 (B) and 1 h (B’). The neonate larvae of *ΔGR66-2* released between the artificial diet with mulberry leaf powder and the artificial diet with a 1:1 ratio of soybean powder to corn powder after 0 (C) and 1 h (C’). Scale bars: 10 mm in A, A’, B, B’, C, and C’. Each assay was performed in triplicate (technical replicates). GR66, gustatory receptor 66; WT, wild-type.
(TIF)

S6 Fig Electrophysiological responses to sucrose and myo-inositol in lateral sensilla and caffeine and salicin in medial sensilla. (A) Representative spike traces of the lateral sensilla of the indicated genotypes stimulated with 2 mM KCl, 10 mM sucrose, and 10 mM myo-inositol. *ΔGR66-2* mutant larvae responded normally to sucrose and inositol. (B) Electrophysiological response frequencies of the lateral sensilla of the indicated genotypes stimulated with 10 mM sucrose and 10 mM myo-inositol. (C) Representative spike traces of medial sensilla of the indicated genotypes stimulated with 2 mM KCl, 10 mM caffeine, and 10 mM salicin. *ΔGR66-2* mutant larvae responded normally to sucrose and inositol. (D) Electrophysiological response frequencies of the medial sensilla of the indicated genotypes stimulated with 10 mM caffeine and 10 mM salicin. (E) Electrophysiological response frequencies of the medial sensilla of the indicated genotypes stimulated with different concentrations of caffeine. (F) Electrophysiological response frequencies of the medial sensilla of the indicated genotypes stimulated with different concentrations of salicin. The data shown were the mean ± SEM (*n* = 20 silkworms). Significance was assessed by a two-tailed *t*-test: ns (not significant). Underlying data can be found in S1 Data. GR66, gustatory receptor 66; SEM, standard error of the mean.
(TIF)

S1 Table Primers used in this work. (DOCX)

S1 Data Numerical data used in the figures. (XLSX)

S1 Movie *ΔGR66-2* mutant fifth-instar larva fed on leaves of mulberry. GR66, gustatory receptor 66.
(AVI)

S2 Movie *ΔGR66-2* mutant fifth-instar larva fed on leaves of Mongolian oak. GR66, gustatory receptor 66.
(AVI)

S3 Movie *ΔGR66-2* mutant fifth-instar larva fed on fruits of apple. GR66, gustatory receptor 66.
(AVI)

S4 Movie *ΔGR66-2* mutant fifth-instar larva fed on fruits of pear. GR66, gustatory receptor 66.
(AVI)

S5 Movie *Δ*GR66-2 mutant fifth-instar larva fed on seeds of soybean. GR66, gustatory receptor 66.
(AVI)

S6 Movie *ΔGR66-2* mutant fifth-instar larva fed on seeds of corn. GR66, gustatory receptor 66.
(AVI)

## Abbreviations

ANOVA: analysis of variance
cDNA: complementary DNA
CRISPR/Cas9: clustered regularly interspaced short palindromic repeats/CRISPR-associated protein-9 nuclease
DMEM: Dulbecco's modified Eagle’s medium
DNase I: deoxyribonuclease I
FBS: fetal bovine serum
EGFP: enhanced green fluorescent protein
GPCR: G-protein-coupled receptor
GR: gustatory receptor
GRN: gustatory receptor neuron
GR66: gustatory receptor 66
HEK293T: human embryonic kidney 293T
L5D3: third-day fifth-instar
*Nos-Cas9*: *pBac[IE1-DsRed2-Nos-Cas9]*
OR: olfactory receptor
ORF: open reading frame
qRT-PCR: quantitative real-time PCR
SAPID: spike analysis programs for insect data
SEM: standard error of the mean
sgRNA: small guide RNA
TALEN: transcription activator-like effector nuclease
*U6-sgRNA*: *pBac[IE1-EGFP-U6-BmGR66-sgRNA]*
WT: wild-type
ZFN: zinc-finger nuclease
