# Supplementary figures and images for: A determining factor for insect feeding preference in the silkworm, Bombyx mori
Source: PLoS Biol. 2019 Feb 27;17(2):e3000162. doi: 10.1371/journal.pbio.3000162 (PMC6411195; doi:10.1371/journal.pbio.3000162)

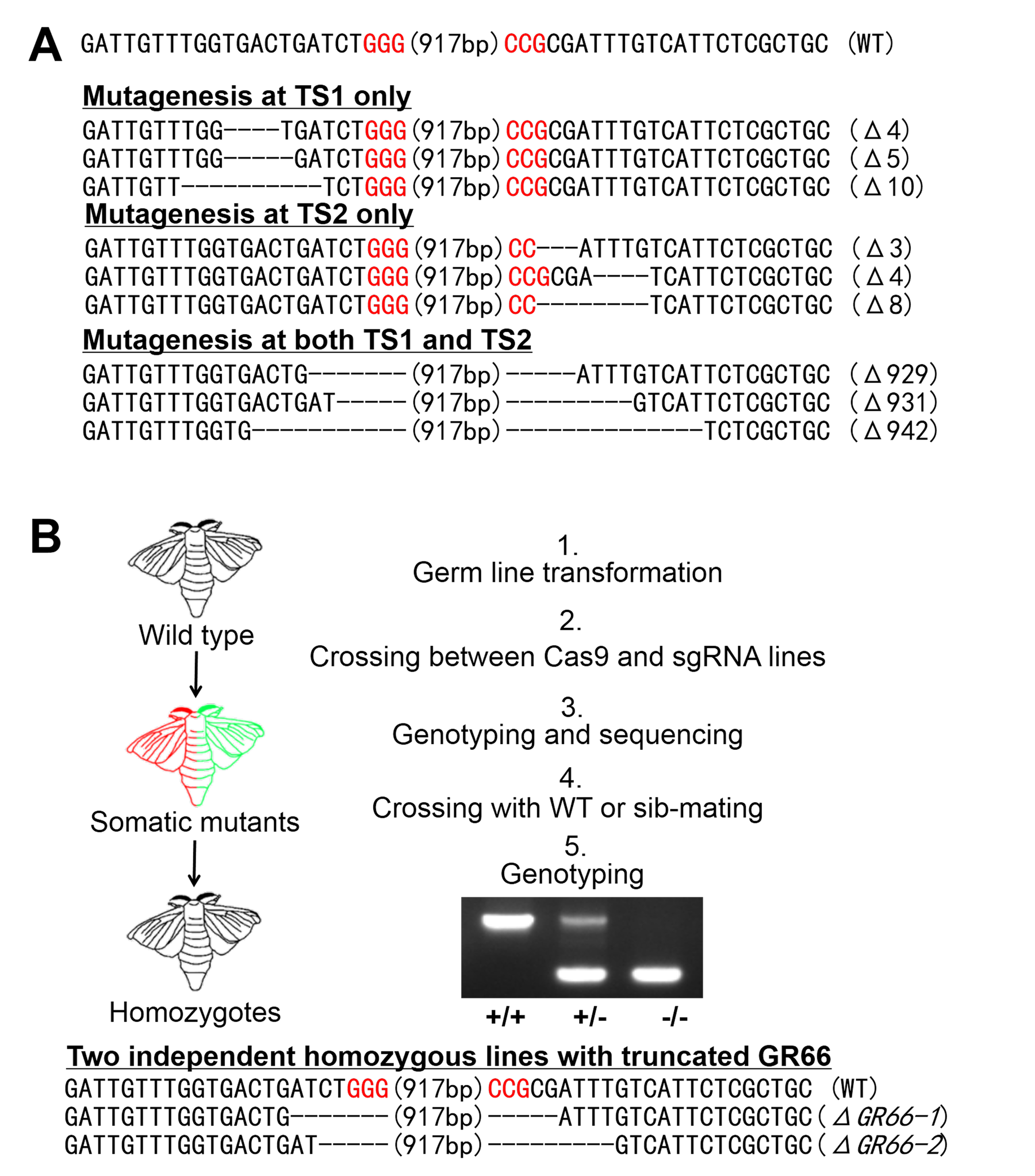

Supplement: S1 Fig — (A) Genomic mutagenesis induced by the transgenic CRISPR/Cas9 system. Various deletion mutations of TS1 and TS2 were detected in heterozygous Nos-Cas9:U6-sgRNA offspring. The numbers in brackets in the middle of each sequence refer to the 1,403-bp interspace fragment that was found between the targeting sites. The PAM sequence is shown in red. (B) The strategy for generation of a homozygous mutant using the transgenic CRISPR/Cas9 system. (1) Preblastoderm silkworm embryos were injected with the transgenic plasmids Nos-Cas9 or U6-sgRNA to produce two transgenic silkworm lines. (2) Subsequently, the two transgenic lines were hybridized to produce founder animals (F1), which expressed both Cas9 and GR66 sgRNAs. (3) The F1 somatic mutant was backcrossed with WT to obtain F2 progeny. The F2 progeny that lacked fluorescence and complete deletion events were backcrossed with WT moths again to obtain F3 animals that were 50% heterozygotes and 50% WT animals. (4) The F3 heterozygous animals were then sib-mated to obtain F4 hybrids that were 25% F4 homozygous mutants, 50% heterozygous mutants, and 25% WT animals. (5) The F4 homozygous mutants were then sib-mated to obtain 100% homozygous F5 progeny, which were used in subsequent experiments. Two GR66 allele mutant lines were established. The sequence below shows the mutation event. The PAM sequence is shown in red. CRISPR/Cas9, clustered regularly interspaced short palindromic repeats/CRISPR-associated protein-9 nuclease; GR66, gustatory receptor 66; Nos-Cas9, pBac[IE1-DsRed2-Nos-Cas9]; PAM, protospacer adjacent motif; sgRNA, small guide RNA; U6-sgRNA, pBac[IE1-EGFP-U6-BmGR66-sgRNA]; WT, wild-type. (TIF) [file pbio.3000162.s001.tif]

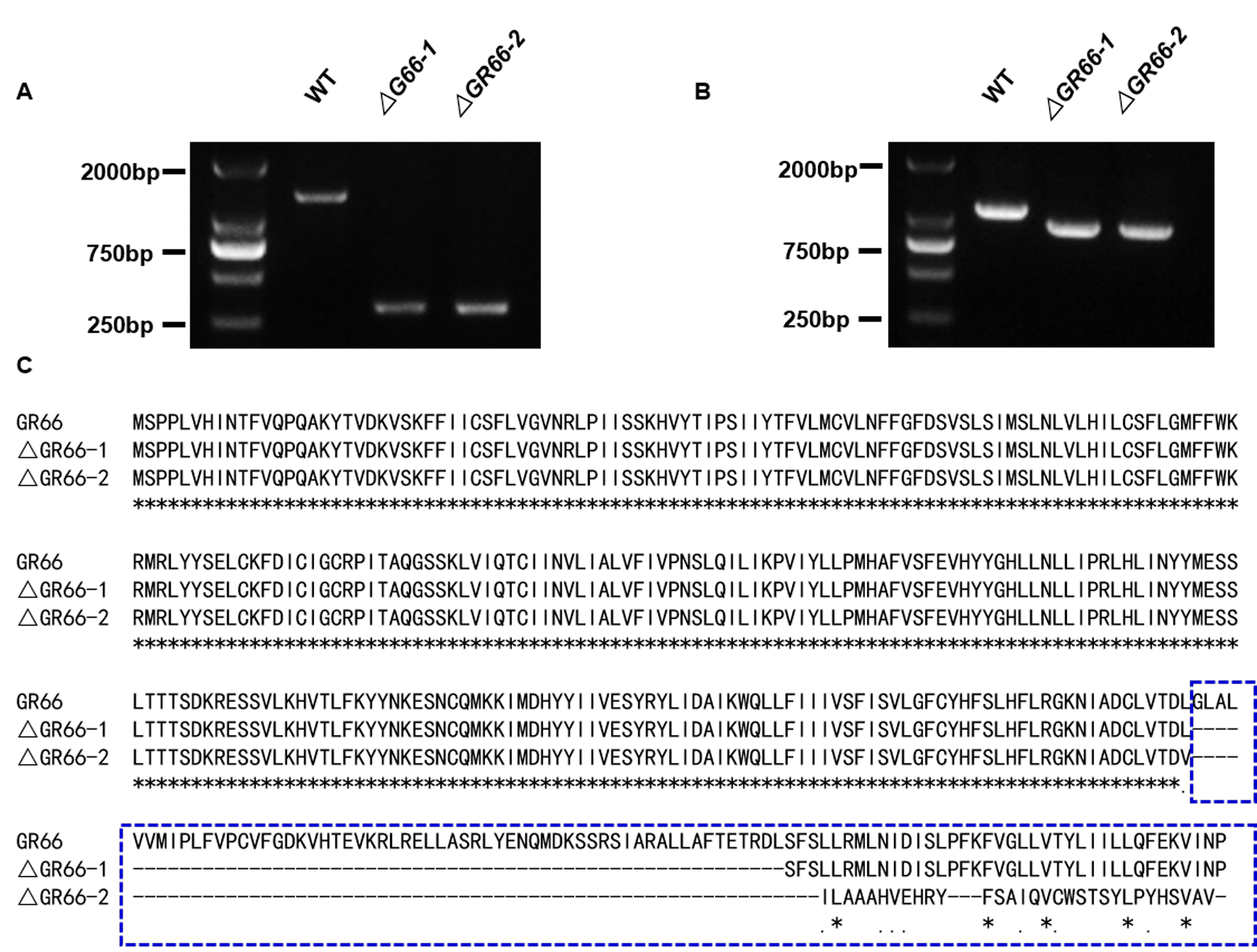

Supplement: S2 Fig — (A) Genomic PCR of GR66 of WT, ΔGR66-1, and ΔGR66-2. ΔGR66-1 had a 929-bp genomic DNA deletion, and ΔGR66-2 had a 931-bp genomic DNA deletion at the GR66 locus. (B) RT-PCR of GR66 of WT, ΔGR66-1, and ΔGR66-2. ΔGR66-1 had a 180-bp deletion, and ΔGR66-2 had a 182-bp deletion in the ORF. (C) Amino acid sequence alignment of the GR66 protein in WT, ΔGR66-2, and ΔGR66-2. ΔGR66-1 is 60 aa shorter than the WT GR66 protein. ΔGR66-2 is 67 aa shorter than the WT GR66 protein. Identical amino acids are indicated with “*.” GR66, gustatory receptor 66; ORF, open reading frame; RT-PCR, reverse transcription-PCR; WT, wild-type. (TIF) [file pbio.3000162.s002.tif]

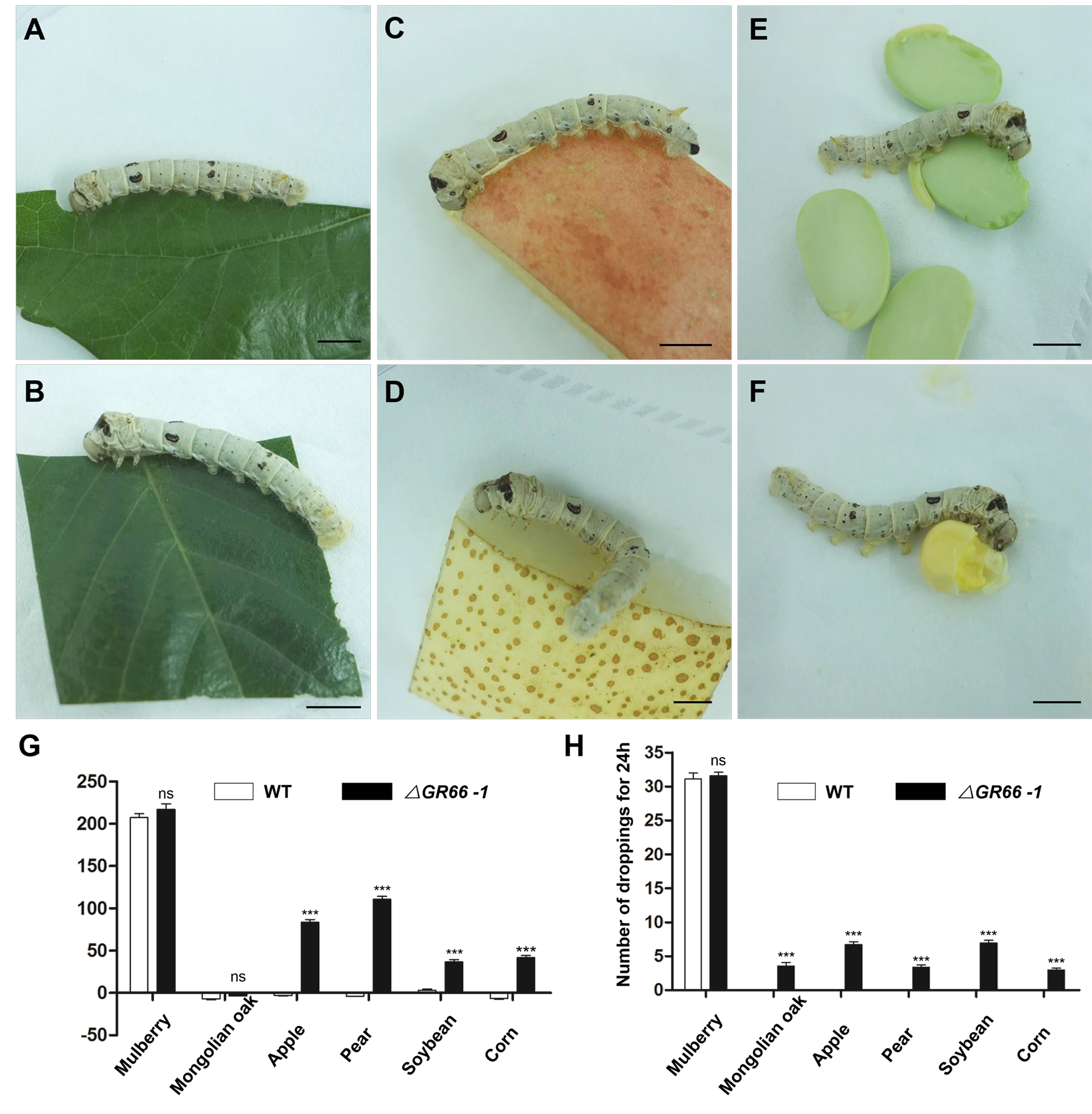

Supplement: S3 Fig — The newly moulted fifth-instar larvae of ΔGR66-1 after 24 h of starvation ate mulberry leaves (A), Mongolian oak (B), apple (C), pear (D), soybean (E), and corn (F). (G) ΔGR66-1 fed on apple, pear, soybean, and corn showed a significant increase in weight when compared to WT fed on the same materials. Scale bars: 5 mm in A, B, C, D, E, and F. (G) ΔGR66-1 fed on apple, pear, soybean and corn showed a significant increase in weight when compared to WT fed on the same materials. The body weights of the larvae of WT and ΔGR66-1 fed on Mongolian oak did not show an increase in weight after 24 h of feeding. (H) Number of droppings (per larva) from larvae fed on mulberry, Mongolian oak, apple, pear, soybean, and corn at 24 h after initiation of feeding. The data shown was the mean ± SEM (n = 18 silkworms). The asterisks indicated significant differences as calculated by a two-tailed t-test: ns (not significant), ***P < 0.001. Underlying data can be found in S1 Data. GR66, gustatory receptor 66; SEM, standard error of the mean; WT, wild-type. (TIF) [file pbio.3000162.s003.tif]

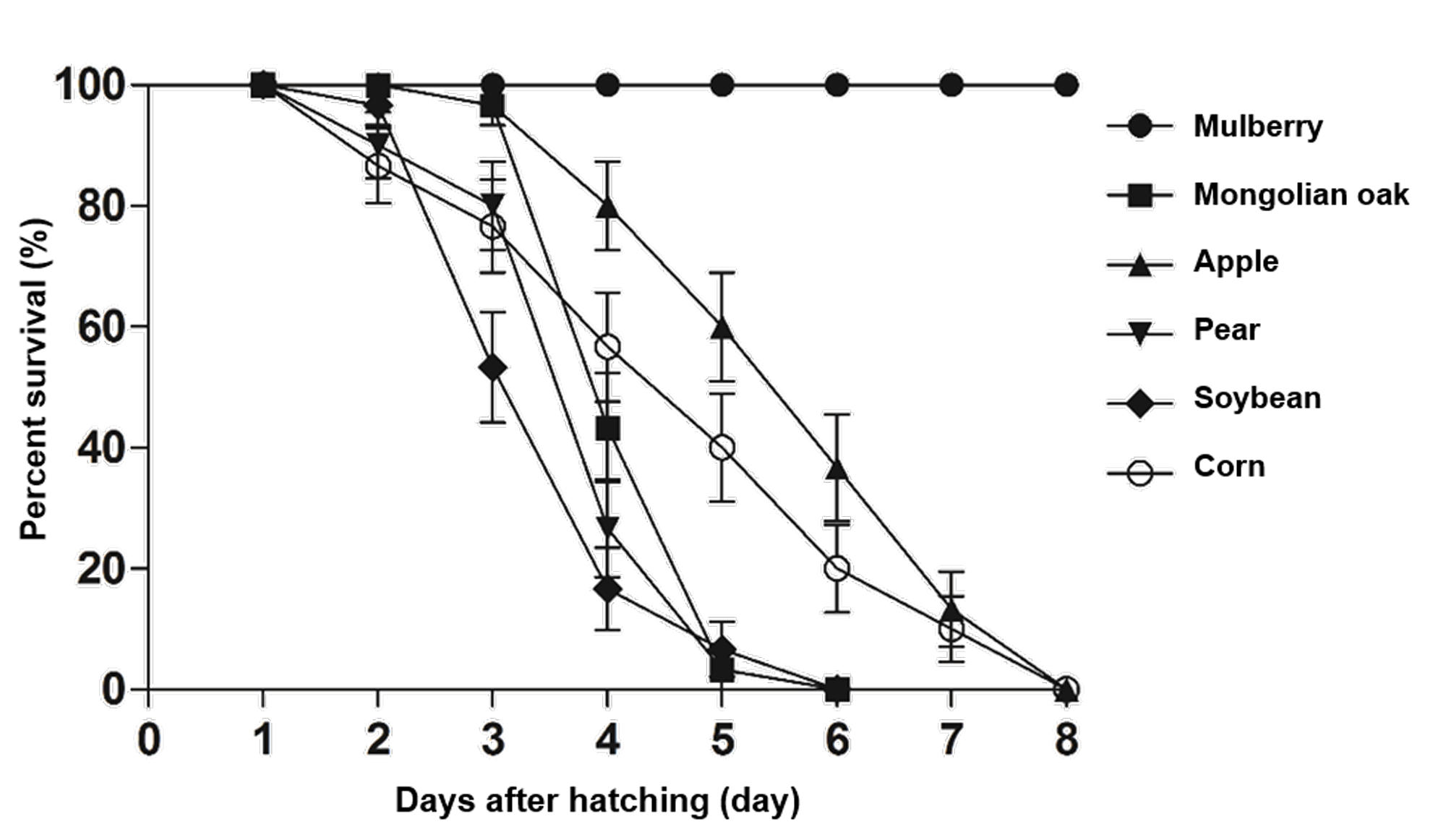

Supplement: S4 Fig — The data shown was the mean ± SEM (n = 30 silkworms). Each assay was performed in triplicate. Underlying data can be found in S1 Data. GR66, gustatory receptor 66; SEM, standard error of the mean. (TIF) [file pbio.3000162.s004.tif]

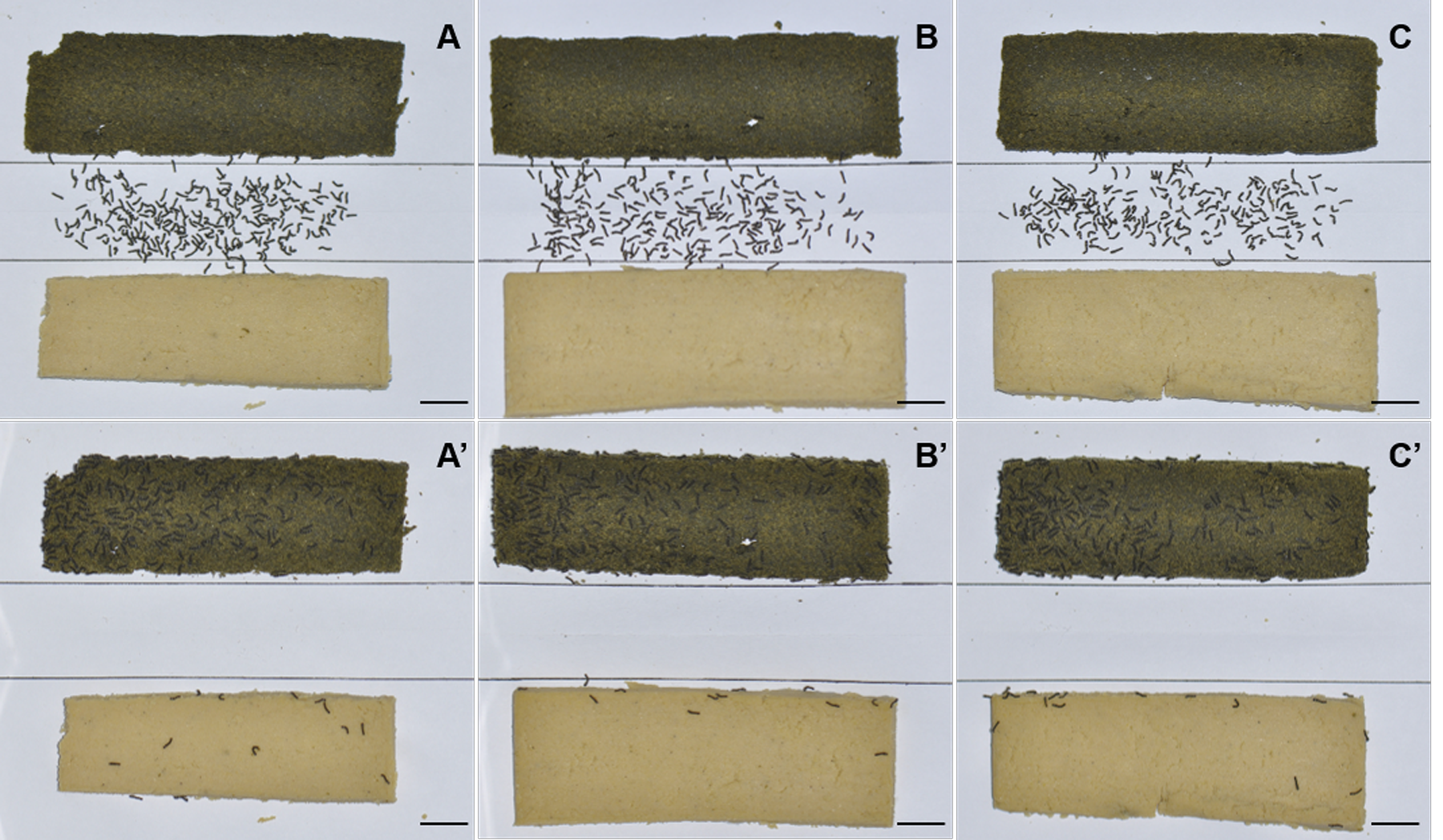

Supplement: S5 Fig — The neonate larvae of WT released between the artificial diet with mulberry leaf powder and the artificial diet with a 1:1 ratio of soybean powder to corn powder after 0 (A) and 1 h (A’). The neonate larvae of ΔGR66-1 released between the artificial diet with mulberry leaf powder and the artificial diet with a 1:1 ratio of soybean powder to corn powder after 0 (B) and 1 h (B’). The neonate larvae of ΔGR66-2 released between the artificial diet with mulberry leaf powder and the artificial diet with a 1:1 ratio of soybean powder to corn powder after 0 (C) and 1 h (C’). Scale bars: 10 mm in A, A’, B, B’, C, and C’. Each assay was performed in triplicate (technical replicates). GR66, gustatory receptor 66; WT, wild-type. (TIF) [file pbio.3000162.s005.tif]

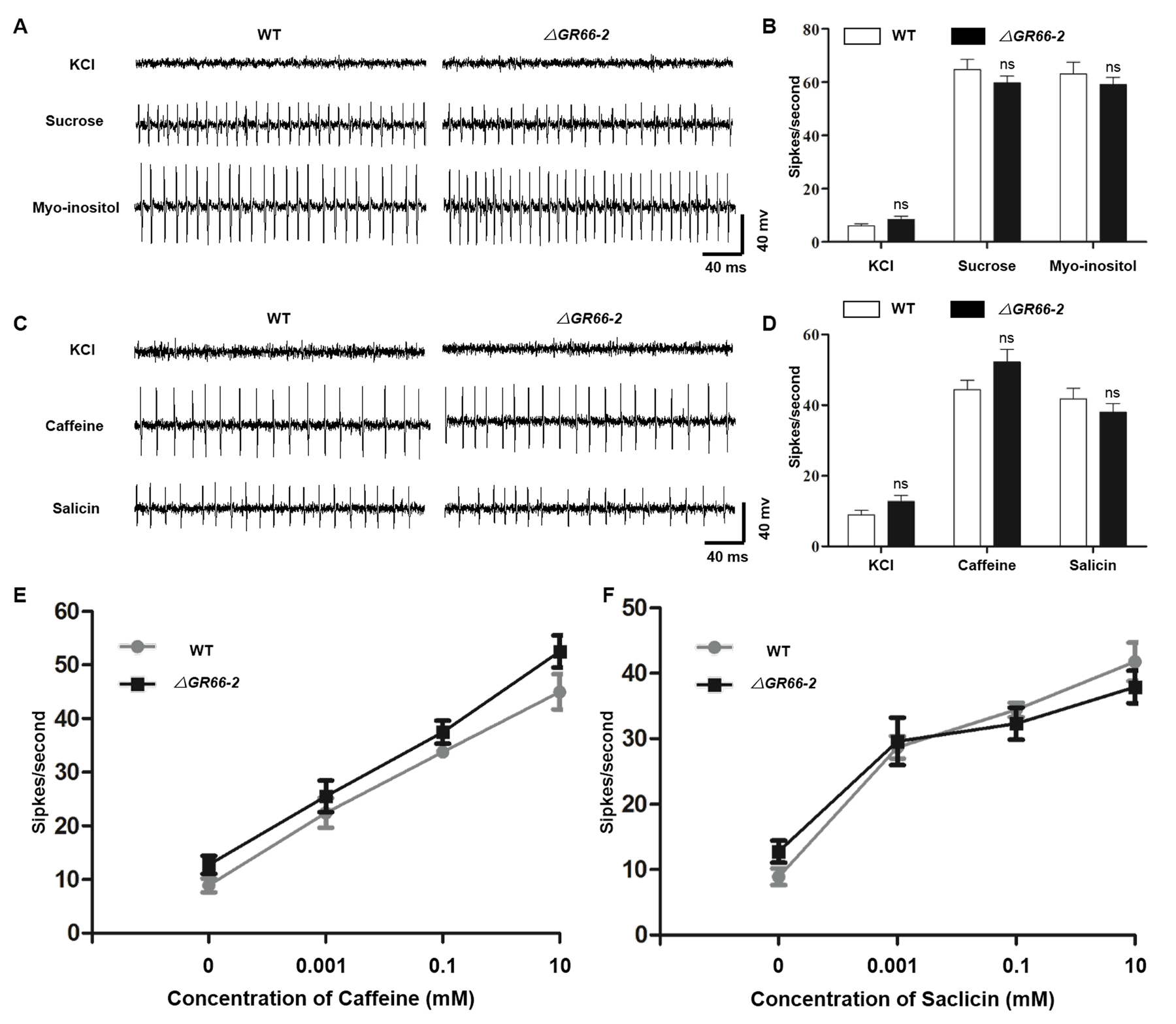

Supplement: S6 Fig — (A) Representative spike traces of the lateral sensilla of the indicated genotypes stimulated with 2 mM KCl, 10 mM sucrose, and 10 mM myo-inositol. ΔGR66-2 mutant larvae responded normally to sucrose and inositol. (B) Electrophysiological response frequencies of the lateral sensilla of the indicated genotypes stimulated with 10 mM sucrose and 10 mM myo-inositol. (C) Representative spike traces of medial sensilla of the indicated genotypes stimulated with 2 mM KCl, 10 mM caffeine, and 10 mM salicin. ΔGR66-2 mutant larvae responded normally to sucrose and inositol. (D) Electrophysiological response frequencies of the medial sensilla of the indicated genotypes stimulated with 10 mM caffeine and 10 mM salicin. (E) Electrophysiological response frequencies of the medial sensilla of the indicated genotypes stimulated with different concentrations of caffeine. (F) Electrophysiological response frequencies of the medial sensilla of the indicated genotypes stimulated with different concentrations of salicin. The data shown were the mean ± SEM (n = 20 silkworms). Significance was assessed by a two-tailed t-test: ns (not significant). Underlying data can be found in S1 Data. GR66, gustatory receptor 66; SEM, standard error of the mean. (TIF) [file pbio.3000162.s006.tif]
