# Supplementary material for: A determining factor for insect feeding preference in the silkworm, Bombyx mori
Source: PLoS Biol. 2019 Feb 27;17(2):e3000162. doi: 10.1371/journal.pbio.3000162 (PMC6411195; doi:10.1371/journal.pbio.3000162)
Supplement: S1 Table — (DOCX) [file pbio.3000162.s007.docx]

**S1 Table. Primers used in this work.**

| Primer name | Primer sequence (5’ to 3’) | Primer purpose |
| --- | --- | --- |
| HR-F | TCACTATAGGGCGAATTGGAGGTTATGTAGTACACATTGTTGTA | Construction of transgenic plasmid |
| *U6*-R1 | AGATCAGTCACCAAACAATCACTTGTAGAGCACGATATTTTGTAT | Construction of transgenic plasmid |
| *sgRNA*-F1 | GATTGTTTGGTGACTGATCTGTTTTAGAGCTAGAAATAGCAAGTT | Construction of transgenic plasmid |
| Overlap-R | CCGCGGAGTCAATGGCTAGCAAAAAAGCACCGACTCGGTG | Construction of transgenic plasmid |
| Overlap-F | GCTAGCCATTGACTCCGCGGAGGTTATGTAGTACACATTGTTGTA | Construction of transgenic plasmid |
| *U6*-R2 | CGATTTGTCATTCTCGCTGCACTTGTAGAGCACGATATTTTGTAT | Construction of transgenic plasmid |
| *sgRNA*-F2 | GCAGCGAGAATGACAAATCGGTTTTAGAGCTAGAAATAGCAAGTT | Construction of transgenic plasmid |
| HR-R | TTTCTTGTTATAGATATCAAAAAAAGCACCGACTCGGTG | Construction of transgenic plasmid |
| TS1-F | AGCGTTATGATACATTTCTGGGC | Identification of mutations |
| TS1-R | TGTCAACTGCATGTCTTGAGG | Identification of mutations |
| TS2-F | AAATGTGTTGAACGCGAGCT | Identification of mutations |
| TS2-R | CAGTTTCTGTGACTTCGTTACCA | Identification of mutations |
| GR66-QF | CGCTGATTGTTTGGTGACTG | qRT-PCR |
| GR66-QR | TCCGTGAAGGCTAGAAGAGC | qRT-PCR |
| RP49-QF | TCAATCGGATCGCTATGACA | qRT-PCR |
| RP49-QR | ATGACGGGTCTTCTTGTTGG | qRT-PCR |
| GR66-RT-F | ATGTCACCACCGCTAGTCCA | RT-PCR |
| GR66-RT-R | CTACGGATTAATAACTTTTTCAAACTGC | RT-PCR |
| KpnI-GR66-F | CGCGGTACCATGTCACCACCGCTAGTC | Construction of recombinant plasmid |
| XbaI-GR66-R | CATGTCTAGACTACGGATTAATAACTTTTTCAAACTGC | Construction of recombinant plasmid |
| KpnI-GFP-F | CGCGGTACCATGGTGAGCAAGGGCGAG | Construction of recombinant plasmid |
| Lap-GFP-R | GACATTGAGCCGCCGCCGCCCTTGTACAGCTCGTCCATG | Construction of recombinant plasmid |
| Lap-GR66-F | GGCGGCGGCGGCTCAATGTCACCACCGCTAGTC | Construction of recombinant plasmid |
| NotI-GR66-R | TTTTCCTTTTGCGGCCGCCTACGGATTAATAACTTTTTCAAAC | Construction of recombinant plasmid |
